# Supplementary material for: Design, Synthesis, and Biological Evaluation of N-Acylhydrazones and Their Activity Against Leishmania amazonensis Promastigotes
Source: Microorganisms. 2025 Jul 2;13(7):1563. doi: 10.3390/microorganisms13071563 (PMC12298860; doi:10.3390/microorganisms13071563)
Supplement: Supplementary file 1 [file microorganisms-13-01563-s001.zip › HPLC amostras-20241114T144635Z-001.zip!/HPLC amostras/HPLC composto 29 - PMM 90-17.pdf]

Farmanguinhos - Fiocruz
Serviço de Métodos Analíticos

PMM 90-17 A Rep.1.lcd
2018 - Síntese 1 - João Mafra - 2-8/2-9-6 - PMM 90-17 A Rep.1.lcd
31/07/2018, 14:05:56

Sample Name	: PMM 90-17 A Rep.1		
Sample ID	: 121164		
Sample Full Path	: 2018 - Síntese 1 - João Mafra - 2-8/2-9-6 - PMM 90-17 A Rep.1.lcd		
Data Filename	: PMM 90-17 A Rep.1.lcd		
Method Filename	: Nitroimidazol.lcm		
Batch File	: 2018 - Síntese 1 - João Mafra - CLAE-11 - CLAE 11_2018 _0731.lcb		
Vial #	: 1-4	Sample Type	: Unknown
Injection Volume	: 10 uL		
Date Acquired	: 31/07/2018 14:06:25	Acquired by	: Licinio Fontoura
Date Processed	: 01/08/2018 10:35:27	Processed by	: Licinio Fontoura
Data Comment	:		

Preparação da amostra

A 1,18mg de amostra foram adicionados 2000µL de metanol, agitado em vortex e levada ao ultrasom por 1 minuto (Conc.de 0,59mg/mL). Posteriormente foi filtrada em membrana PVDF de 0,22µm e injetada.

Method

<<Header>>

<<Pump>>
Total Flow : 1,0000 mL/min
Pump B : LC-20ADXR
Pump A : LC-20ADXR

<<Mobile Phase Name>>
Pump A Mobile Phase A : Água
Pump B Mobile Phase A : Acetonitrila

<<LC Time Program>>

Time	Module	Command	Value	Comment
0,01	Pumps	Pump B Conc.	20	
5,00	Pumps	Pump B Conc.	20	
22,00	Pumps	Pump B Conc.	90	
25,00	Pumps	Pump B Conc.	90	
33,00	Pumps	Pump B Conc.	20	
39,00	Pumps	Pump B Conc.	20	
39,01	Controller	Stop		

<<Data Acquisition>>
LC Stop Time : 39,01 min

<<Autosampler>>
Cooler Temperature : Off

<<Oven>>
Oven Temperature : 30 C

<<Column Infomation>>
Column Name : Supelcosil LC-8 5µm
Column ID : CL0173
Column Length : 150 mm
Inner Diameter : 4,6 mm

Instrumentation

<<Instrument>>

<<Pump A>>
Pump A Type : LC-20ADXR

<<Pump B>>
Pump B Type : LC-20ADXR

<<System Controller>>
SCL Type : CBM-20A

<<Autosampler>>
Autosampler : SIL-30AC

<<Oven>>
Oven Type : CTO-20AC

<<Column>>
Column Name : Supelcosil LC-8 5µm
Column ID : CL0173
Length : 150 mm
Internal Diameter : 4,6 mm
Max Number of Injections : 100000
Expiration Date : 29/04/2025
Max Temperature : 60,0 C
Installation Date : 14/03/2018

<<PDA>>
Detector Name : PDA
Detector Type : SPD-M20A

<<Degasser>>
Degasser : DGU-20A5R

Farmanguinhos - Fiocruz
Serviço de Métodos Analíticos

PMM 90-17 A Rep.1.lcd
2018 - Síntese 1 - João Mafra - 2-8/2-9-6 - PMM 90-17 A Rep.1.lcd
31/07/2018, 14:05:56

Chromatogram

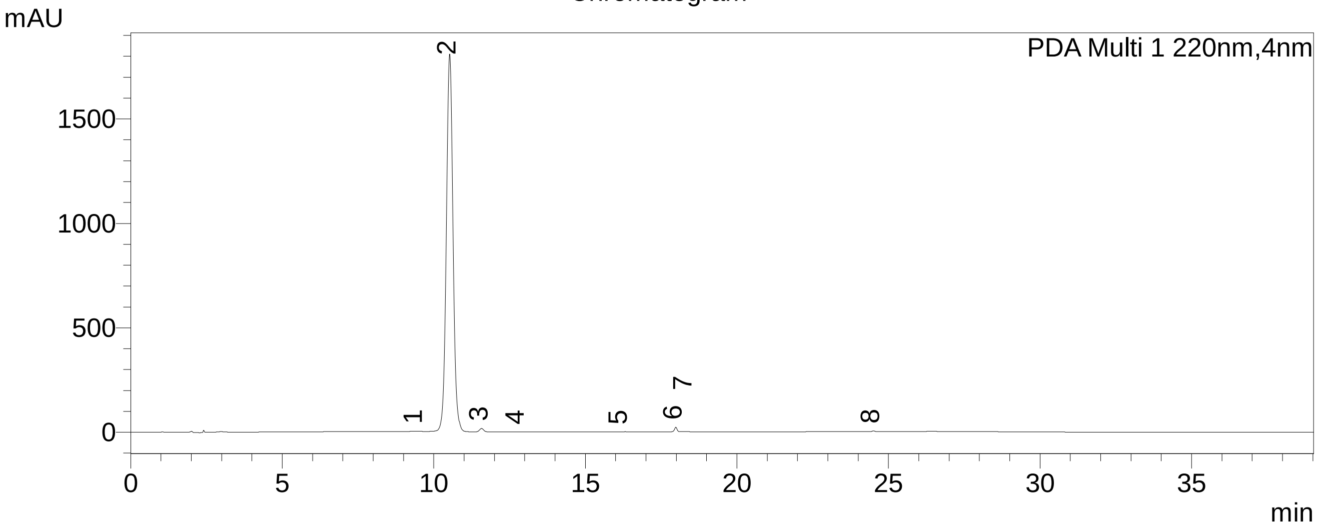

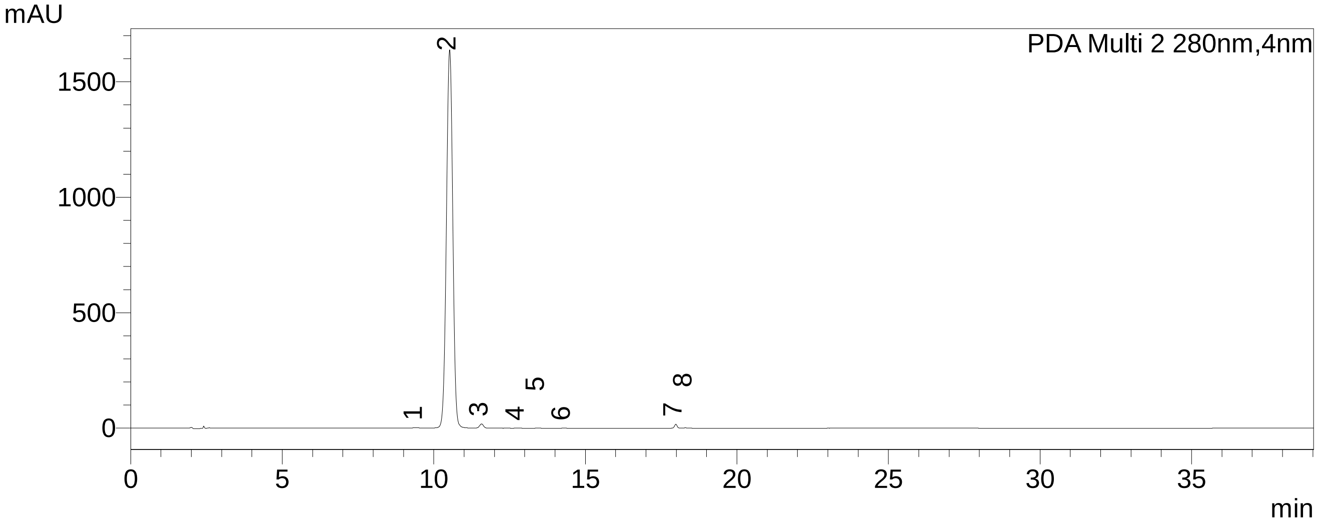

Peak Table

PDA Ch1 220nm

Peak#	Ret. Time	Name	Area	Area%	Theoretical Plates/meter(USP)	Tailing Factor	Resolution(USP)	Capacity Factor(k')
1	9,42		39308	0,1	31048	0,676	--	--
2	10,53		27803486	98,5	71920	1,041	2,315	0,118
3	11,58		158742	0,6	209672	1,028	3,155	0,229
4	12,78		9825	0,0	313620	1,012	4,825	0,357
5	16,18		14440	0,1	3621	--	2,260	0,718
6	17,99		140461	0,5	1033663	1,014	1,219	0,910
7	18,31		27754	0,1	279360	--	1,199	0,944
8	24,50		19253	0,1	2316286	0,970	23,641	1,602
Total			28213269	100,0				

PDA Ch2 280nm

Peak#	Ret. Time	Name	Area	Area%	Theoretical Plates/meter(USP)	Tailing Factor	Resolution(USP)	Capacity Factor(k')
1	9,42		23368	0,1	47624	0,964	--	--
2	10,53		24054740	98,6	77429	0,991	2,640	0,117
3	11,58		168178	0,7	213457	0,992	3,240	0,229
4	12,78		9892	0,0	285187	1,024	4,749	0,356
5	13,44		9208	0,0	412000	1,042	2,854	0,427

Farmanguinhos - Fiocruz
Serviço de Métodos Analíticos

PMM 90-17 A Rep.1.lcd
2018 - Síntese 1 - João Mafra - 2-8/2-9-6 - PMM 90-17 A Rep.1.lcd
31/07/2018, 14:05:56

Peak#	Ret. Time	Name	Area	Area%	Theoretical Plates/meter(USP)	Tailing Factor	Resolution(USP)	Capacity Factor(k')
6	14,30		5849	0,0	418817	0,998	3,854	0,517
7	17,99		102338	0,4	1081716	0,958	18,135	0,909
8	18,31		12042	0,0	427683	1,164	1,375	0,943
Total			24385616	100,0				
